# Annual Meeting of the Erie County Medical Society

**Published:** 1864-01

**Authors:** Leon F. Harvey

**Affiliations:** Secretary


### ANNUAL MEETING OF THE ERIE COUNTY MEDICAL SOCIETY.

This Society held its Annual Meeting at the rooms of the Buffalo Medical Association Tuesday, January 12th, 1864. The election was held, which resulted as follows:

| | |
|---|---|
| President, - - - - | Dr. C. C. Wyckoff. |
| Vice-President, - - - | Dr. Geo. Abbott. |
| Secretary, - - - - | Dr. Leon F. Harvey. |
| Treasurer, - - - | Dr. William Ring. |
| Librarian, - - - - | Dr. J. C. Samo. |

Primary Board.—Dr. C. S. Dayton, Dr. S. W. Wetmore, Dr. P. P. E. Tobie.

Censors.—Dr. T. M. Johnson: Anatomy, Physiology, and Surgery. Dr. M. H. Shaw: Practical Medicine and Obstetrics. Dr. J. R. Lothrop: Chemistry and Pharmacy. Dr. O. K. Parker: Materia Medica and Botany. Dr. J. E. Peters: Medical Jurisprudence and Pathology.

It was resolved by the Society that notices be served upon all not members in the County, to become members of this Society, and if failing to do so, they be treated according to the reading of the By-Laws of the Society.

Dr. J. A. Peters read an able paper, the subject of which was Quinine. He was given a vote of thanks, and a copy was requested for publication in the Buffalo Medical and Surgical Journal. Dr. Winne stated to the Society that the case of Dr. Bartlett, which was decided against the Society in the Supreme Court, had been carried to the Court of Appeals, and a decision would be rendered in February, probably favorable to the Society.

Drs. George Ayer and H. B. Horton were admitted as members.

Leon F. Harvey, Secretary.

